# High-*T*_*c*_ superconductor Fe(Se,Te) monolayer: an intrinsic, scalable and electrically tunable Majorana platform

**DOI:** 10.1093/nsr/nwab087

**Published:** 2021-05-19

**Authors:** Xianxin Wu, Xin Liu, Ronny Thomale, Chao-Xing Liu

**Affiliations:** Institut für Theoretische Physik und Astrophysik, Julius-Maximilians-Universität Würzburg, 97074 Würzburg, Germany; School of Physics, Huazhong University of Science and Technology, Wuhan 430074, China; Wuhan National High Magnetic Field Center, Huazhong University of Science and Technology, Wuhan 430074, China; Institut für Theoretische Physik und Astrophysik, Julius-Maximilians-Universität Würzburg, 97074 Würzburg, Germany; Department of Physics, the Pennsylvania State University, University Park, PA 16802, USA

**Keywords:** high-order topological superconductivity, Majorana zero modes, iron-based superconductors

## Abstract

Iron-based superconductors have been identified as a novel platform for realizing Majorana zero modes (MZMs) without heterostructures, due to their intrinsic topological properties and high-*T*_*c*_ superconductivity. In the two-dimensional limit, the FeTe_1−*x*_Se_*x*_ monolayer, a topological band inversion has recently been experimentally observed. Here, we propose to create MZMs by applying an in-plane magnetic field to the FeTe_1−*x*_Se_*x*_ monolayer and tuning the local chemical potential via electric gating. Owing to the anisotropic magnetic couplings on edges, an in-plane magnetic field drives the system into an intrinsic high-order topological superconductor phase with Majorana corner modes. Furthermore, MZMs can occur at the domain wall of chemical potentials at either one edge or certain type of tri-junction in the two-dimensional bulk. Our study not only reveals the FeTe_1−*x*_Se_*x*_ monolayer as a promising Majorana platform with scalability and electrical tunability and within reach of contemporary experimental capability, but also provides a general principle to search for realistic realization of high-order topological superconductivity.

## Teaser

The authors propose to create Majorana zero modes by applying an in-plane magnetic field to high T_c_ Fe(Te,Se) monolayer, originating from a higher-order topology, which indicates that Fe(Te,Se) is a promising Majorana platform with scalability and electrical tunability.

## INTRODUCTION

Within the nomenclature of condensed matter, a Majorana zero mode (MZM) is an anyonic quasi-particle excitation with non-abelian statistics, which underpins the concept of topological quantum computations [1–5]. A variety of physical systems have been theoretically proposed to realize MZMs, including the ν = 5/2 fractional quantum Hall state [4,6,7], a chiral *p*-wave state possibly realized in Sr_2_RuO_4_ superconductors (SCs) [8,9], semiconducting nanowires in proximity to SCs subject to magnetic fields [10,11], the surface of topological insulators (TIs) in proximity to SCs [12], quantum anomalous Hall insulator-SC heterostructures [13,14] and ferromagnetic atomic chains on SCs [15,16]. Major experimental efforts currently focus on heterostructures made of SCs and spin-orbit coupled systems (such as TIs or semiconducting nanowires), in which evidence of MZMs has been found [17–23]. Unambiguous detection and manipulation of MZMs in these heterostructures, however, heavily rely on the SC proximity effect that suffers from the complexity of the interface. Furthermore, the low operation temperature of conventional superconducting materials complicates further manipulation of MZMs. It is thus desirable to find an intrinsic, robust and controllable Majorana platform that is compatible with existing fabrication and patterning technologies. To this end, recent theoretical predictions and the experimental verification of a topological superconductivity (TSC) phase at the surface of Fe(Se,Te) SCs [24–30] provide exciting opportunities due to the intrinsic nature of both the superconductivity and non-trivial band structure that further comes along with a comparably high critical temperature *T*_*c*_. More recently, the direct observation of band inversion in the two-dimensional (2D) Fe(Se,Te) monolayer suggests the coexistence of a quantum spin Hall (QSH) state and superconductivity, thus providing a new 2D platform for MZMs [31,32], with a *T*_*c*_ of 40 K [33] and a large in-plane upper critical field of about 45 T [34].

**Figure 1.**
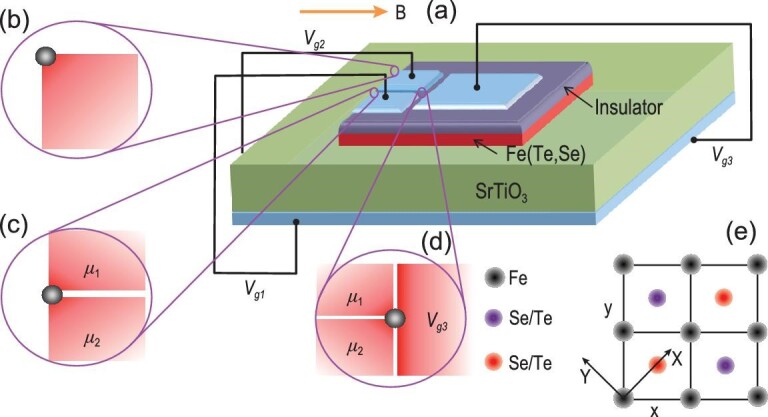
Schematic of the Majorana platform based on an Fe(Se,Te) monolayer (a). MZMs can be found at three different locations: (b) the corner between two perpendicular edges; (c) the CPDW along the 1D edge; (d) the tri-junction in the 2D bulk. Here μ_1, 2_ denote chemical potentials and *V*_*g*3_ is for the asymmetric potential, both of which can be generated by a dual gate voltage. The gray circles in (b), (c) and (d) represent the MZMs, and the magnetic field is in-plane. (e) The crystal structure for the Fe(Te,Se) monolayer and the coordinate system. The gray circles represent Fe atoms and the red (purple) circles represent Se/Te atoms above (below) the Fe layer.

In this work, we theoretically explore different feasible experimental configurations to realize MZMs in an Fe(Se,Te) monolayer by controlling the local chemical potential and the in-plane magnetic field. The experimental setup for an Fe(Te,Se) monolayer with local gating is shown in Fig. 1(a). By studying the topological phase transition (TPT) at the 1D edge and its dependence on the magnetic field direction, we demonstrate the existence of MZMs at the corner of two perpendicular edges with the in-plane magnetic field parallel to one edge (Fig. 1(b)), derived from a magnetic-anisotropy-induced high-order topological superconductor phase, and the chemical potential domain wall (CPDW) along the 1D edge (Fig. 1(c)). The magnetic anisotropy intrinsically originates from the topological band inversion between states at the Γ point with different total angular momenta of the QSH state. We further reveal a 2D bulk TPT between the QSH state and a trivial insulator induced by electric gating in the Fe(Te,Se) monolayer, due to which the MZM can also be trapped in a tri-junction (Fig. 1(d)).

## RESULT

### Model Hamiltonian and the TPT at the 1D edge

We first demonstrate the existence of the TPT at the 1D edge in an Fe(Te,Se) monolayer upon applying an in-plane magnetic field. We start from a tight-binding model including five Fe 3*d* orbitals and spin-orbit coupling (SOC) [25]

(1) \begin{eqnarray*} \mathcal {H}_0&=&\sum _{\alpha \beta }\sum _{mn\sigma }\sum _{ij}(t^{mn}_{\alpha \beta ,ij} +\epsilon _{\alpha }\delta _{mn}\delta _{\alpha \beta }\delta _{ij})\nonumber\\ &&\times\,\, c^{\circ}_{\alpha m\sigma }(i)c_{\beta n \sigma }(j)\nonumber \\ &&+\,\,\sum _{i\alpha \sigma \sigma ^{\prime }} \lambda _{\rm soc} M^{\sigma \sigma ^{\prime }}_{mn} c^{\circ}_{\alpha m\sigma }(i)c_{\alpha n \sigma ^{\prime }}(i), \end{eqnarray*}

where α, β = *A*, *B* labels the sublattices (two Fe atoms in one unit cell in Fig. 1(e)), σ labels the spin, *m*, *n* label five *d* orbitals, and *i*, *j* label the indices of the unit cell. The $t^{mn}_{\alpha \beta ,ij}$ are the hopping parameters, the ε_*m*_ are the on-site energies of Fe *d* orbitals and λ_soc_ labels the SOC strength. The values of these parameters can be found in the online supplementary material (SM). Here $c^{\circ}_{\alpha m \sigma }(i)$ is the creation operator for an electron with spin σ and orbital *m* at the α sublattice site of the unit cell *i*. In order to treat superconductivity in an Fe(Se,Te) monolayer without entering a detailed microscopic derivation, we consider spin singlet intra-orbital pairing within the same sublattice, for which the SC Hamiltonian reads

(2) \begin{eqnarray*} \mathcal {H}_{\rm SC}&=&\sum _{\alpha m\sigma ,ij}\sigma \bigg (\Delta _0\delta _{ij}+\frac{\Delta _1}{4}\delta _{\langle \langle ij \rangle \rangle }\bigg )\nonumber\\ &&\times\,\,c^{\circ}_{\alpha m\sigma }(i)c^{^\circ }_{\alpha m \bar{\sigma }}(j)+{\rm H.c.}, \end{eqnarray*}

where Δ_0_ is the on-site pairing and 〈〈*ij*〉〉 labels the next-nearest-neighbor sites for the pairing parameter Δ_1_, which generates the well-known *s*_±_-wave pairing in iron-based superconductors [35]. Here, we neglect the inter-orbital pairing, which may also exist [36], but will not have any qualitative effect. The Zeeman coupling is given by

(3) \begin{eqnarray*} \mathcal {H}_{Z}&=&\sum _{\alpha mn\sigma \sigma ^{\prime }i}\mu _B g_0 \boldsymbol {B}\cdot \left (\boldsymbol {s}_{\sigma \sigma ^{\prime }}\delta _{mn}+\frac{1}{2}\boldsymbol {L}_{mn}\delta _{\sigma \sigma ^{\prime }} \right)\nonumber\\ &&\times\,\,c^{\circ}_{\alpha m\sigma }(i)c_{\alpha n \sigma ^{\prime }}(i) \end{eqnarray*}

with the Bohr magneton μ_*B*_ and the *g*-factor *g*_0_. Here $\boldsymbol {s}$ and $\boldsymbol {L}$ are spin and orbital angular momentum operators with the forms given in the online SM.

**Figure 2.**
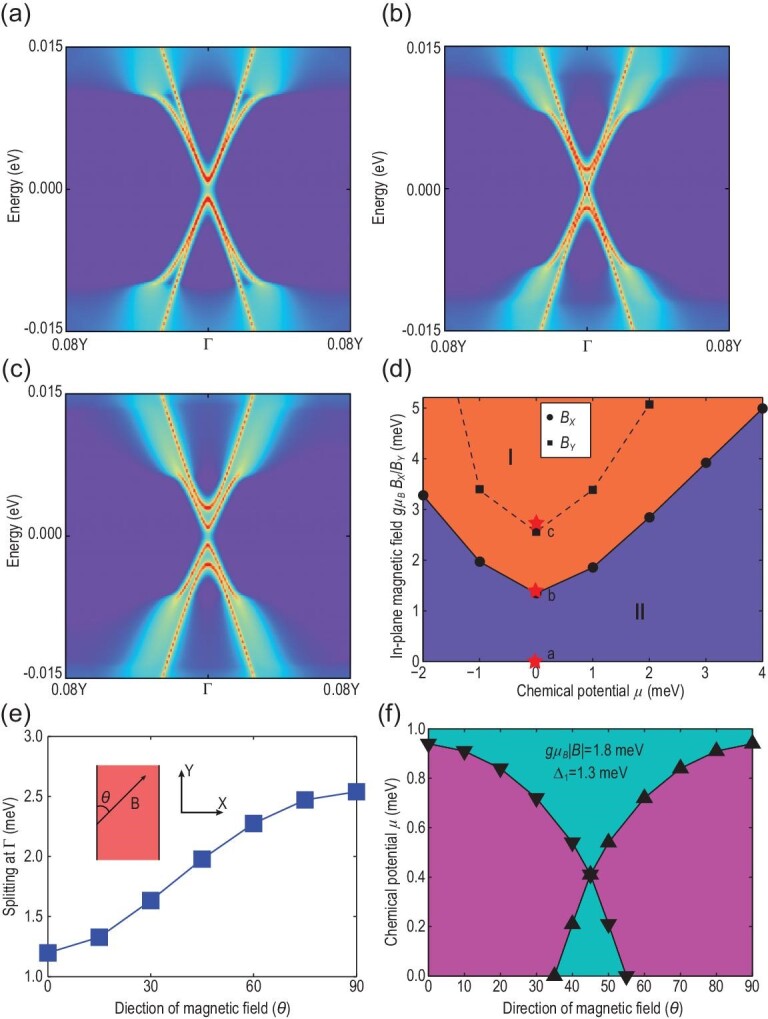
TPT on edges and anisotropic magnetic coupling. Energy spectra of the semi-infinite system with the (100) edge as a function of the in-plane magnetic fields: (a) *B*_*X*_ = 0, (b) *B*_*X*_ = 1.4 meV and (c) *B*_*X*_ = 2.8 meV. (d) TPT as a function of magnetic fields and chemical potentials for the (100) edge. The black circles (squares) correspond to the TPT line for the magnetic field *B*_*X*_ (*B*_*Y*_) perpendicular (parallel) to the edge. The orange and purple regions represent phases I and II, respectively. (e) Zeeman splitting of edge states as a function of the magnetic field angle with respect to the 1D edge (*Y* axis), where *g*μ_*B*_|*B*| is fixed to be 1.8 meV. Inset shows the angle between the magnetic field and 1D edge (thick black line). (f) Phase diagram of the existence regime for MZMs at the corner as a function of the chemical potential μ and magnetic field angle θ. The up(down)-pointing triangles represent the TPT line for (100)((010)) edge. In the pink/blue regimes, the helical edge states at two perpendicular edges have different/the same topological characters, and thus they can/cannot host MZMs at the corner. The superconducting gap Δ_1_ is set to be 1.3 meV and the chemical potential is chosen relative to the Dirac point of edge states.

To study the edge properties of an Fe(Se,Te) monolayer, we consider a semi-infinite system for the above Bogoliubov–de Gennes (BdG)-type tight-binding Hamiltonian $\mathcal {H}_0+\mathcal {H}_{\rm SC}+\mathcal {H}_Z$ for open boundary conditions. We consider the (100) edge (with the open boundary condition along the *X* direction in Fig. 1(e)) and the angle between the magnetic field and the 1D edge is labeled by θ in the inset of Fig. 2(e). Fig. 2(a)–(c) shows the edge energy spectrum along Γ − *Y* for different *X*-directional magnetic fields (θ = 90^°^). Here we adopt the *s*_±_-wave pairing with SC parameters Δ_0_ = 0 and Δ_1_ = 1.3 meV and the magnetic-field-dependent SC gap is assumed to be $\Delta _1(B)=\Delta _1\sqrt{1-{B^2}/{B^2_c}}$ [37], where *B*_*c*_ = 45 T is the upper critical field. We find an SC gap at Γ for helical edge states at zero magnetic field (Fig. 2(a)). An *s*-wave pairing with non-zero Δ_0_ will only have quantitative effects on our results as the edge Dirac cone is located around Γ. Upon increasing the magnetic field to μ_*B*_*g*_0_*B*_*X*_ = 1.4 meV, the Kramers degeneracy at Γ is split due to time reversal symmetry breaking. One branch of the bands close the SC gap at Γ and form a gapless mode with linear dispersion (Fig. 2(b)). The corresponding low-energy effective theory is equivalent to the Kitaev model for 1D *p*-wave spinless SCs (see Sections III and IV of the online SM), suggesting a TPT with 1D gapless Majorana mode occurring at this gap closing. Further increasing the magnetic field (μ_*B*_*g*_0_*B*_*X*_ = 2.8 meV) makes the gap re-open. The magnetic gap dominates over the SC gap (Fig. 2(c)), thus driving the system into a topologically distinct phase from that in Fig. 2(a).

We track the gap evolution as a function of magnetic fields *B* and chemical potentials μ in Fig. 2(d). The band structures in Fig. 2(a)–(c) correspond to the red stars (labeled a, b, and c) in the *B* − μ phase diagram of Fig. 2(d). A gap closing line separates two topologically distinct phases, one dominated by a magnetic gap and the other dominated by the SC gap, labeled as phases I and II in Fig. 2(d). The existence of the TPT at the edge of an Fe(Se,Te) monolayer suggests that the MZMs can exist at the domain wall between phases I and II. This scenario was previously discussed for other QSH systems in proximity to SCs [5,12]. We find a minimal value of the *B* field relating to 1.4 meV, i.e. corresponding to a magnetic field of 12 T, assuming that *g*_0_ = 2, for the TPT line at μ = 0 meV. This minimal value is set by the SC gap and thus a large enough magnetic field is required to achieve phase I. Fortunately, this magnetic field is still well below the in-plane critical magnetic field *B*_*c*_ ∼ 45 T of an Fe(Se,Te) monolayer [34]. The bulk superconducting gap of the Fe(Te,Se) monolayer is much larger than the above adopted gap. However, if we further include the temperature dependence of the gap, a topological phase transition will always occur. The detailed estimation of the critical field can be found in Section III of the online SM.

The magnetic gap further depends on the angle θ in Fig. 2(e). As θ rotates from 0° to 90°, the gap induced solely by Zeeman coupling monotonically increases from 1.1 to 2.5 meV in Fig. 2(e) for a fixed magnetic field amplitude |*g*μ_*B*_*B*| = 1.8 meV and Δ_1_ = 0. The anisotropy of the magnetic gap between perpendicular and parallel magnetic fields is significant, $\delta _M={(V_{\rm max}-V_{\rm min})}/{V_{\rm avg}}\sim 78\%$. As a consequence, the TPT line for the parallel magnetic field (θ = 0°) is different from that of the perpendicular magnetic field (θ = 90°), as shown by the dashed line in Fig. 2(d). We further calculate the TPT lines for the two orthogonal edges (the edges along the *X* and *Y* directions) as a function of the chemical potential μ and the field direction angle θ with a fixed field amplitude |*g*μ_*B*_*B*| = 1.8 meV and Δ_1_ = 1.3 meV, as shown in Fig. 2(f). Topological properties of two edges are the same (distinct) in the blue (pink) regions. The existence of magnetic anisotropy for the edge states is essential for the MZMs at the corner, as discussed below.

### Origin of magnetic anisotropy

We further study the origin of magnetic anisotropy for edge states in the Fe(Te,Se) monolayer. To capture the topological property of the Fe(Se,Te) monolayer, the odd-parity $j_z=\pm \frac{1}{2}$ and even-parity $j_z=\pm \frac{3}{2}$ states at Γ need to be included in the effective Hamiltonian while the even-parity $j_z=\pm \frac{1}{2}$ state is omitted, although it gives the highest valence band [25] (see the analysis in the online SM). On the basis functions $\psi ^{^\circ }_{\boldsymbol {k}}=(c^{^\circ }_{\boldsymbol {k}/{2}},c^{^\circ }_{\boldsymbol {k}{3}/{2}},c^\circ_{\boldsymbol {k} -{1}/{2}},c^\circ_{\boldsymbol {k}-{3}/{2}})$, the effective Hamiltonian takes the form of the Bernevig-Hughes-Zhang (BHZ) model [25,38], given by $\mathcal {H}_{\rm BHZ}=\sum _{\boldsymbol {k}}\psi ^\circ_{\boldsymbol {k}}h_0(\boldsymbol {k})\psi _{\boldsymbol {k}}$ with

(4) \begin{eqnarray*} h_0(\boldsymbol {k})=\epsilon _0({\boldsymbol k}) + M({\boldsymbol k})\sigma _3+A(k_Y s_0\sigma _1\!+\! k_Xs_3\sigma _2),\nonumber\\ \end{eqnarray*}

where $\epsilon _0({\boldsymbol k})=C-D(k^2_X+k^2_Y)$, $M(k)=M-B(k^2_X+k^2_Y)$, *s*_*l*_ and σ_*l*_ label the Pauli matrices in the pseudo-spin and pseudo-orbital spaces and *C*, *D*, *M*, *B*, *A* are material-dependent parameters. The in-plane Zeeman coupling $\mathcal {H}_Z$ can also be projected onto the basis functions $\psi ^\circ_{\boldsymbol {k}}$ and is transformed to *h*_*Z*_ = μ_*B*_(*g*_1_*P*_1/2_ + *g*_2_*P*_3/2_)*B*_*X*_*s*_1_ + μ_*B*_(*g*_1_*P*_1/2_ − *g*_2_*P*_3/2_)*B*_*Y*_*s*_2_, where *P*_1/2(3/2)_ = (σ_0_ + ( − )σ_3_)/2 is the projector operator in the subspace of the *j*_$z$_ = ±1/2(±3/2) states and the *g*_1, 2_ are effective *g*-factors for the BHZ model. To investigate the effective Zeeman coupling of helical edge states, we first calculate the helical edge state from the BHZ Hamiltonian. For the (100) edge, we can omit the $\epsilon _0(\boldsymbol {k})$ term and adopt *k*_*X*_ → −*i*∂_*X*_. The Hamiltonian reads

(5) \begin{eqnarray*} h_0(-i\partial _X,k_Y)&= [M-B(-\partial ^2_X+k^2_Y)]\sigma _3\nonumber \\ &\quad +A(k_Y s_0\sigma _1-i\partial _Xs_3\sigma _2), \end{eqnarray*}

of which the eigenvalue equation *h*_0_Ψ_*p*_(*X*) = *E*_*p*_Ψ_*p*_(*X*) at *k*_*Y*_ = 0 under the boundary condition Ψ_*p*_(*X* → 0) = Ψ_*p*_(*X* → +∞) = 0 can be solved and two zero-energy mode solutions are given by

(6) \begin{eqnarray*} \Psi _p(X)=N\,{\rm sinh}(\eta _1X)e^{\eta _2X}\phi _p, \end{eqnarray*}

where the normalization factor $N=4|\eta _2(\eta _2^2-\eta _1^2)/\eta _1^2|$, $\eta _1=\sqrt{{A^2}/{4B^2}-{M}/{B}}$ and η_2_ = *A*/2*B*. The eigenvectors φ_*p*_ satisfy the eigenequation *s*_3_σ_1_φ_*p*_ = φ_*p*_ and can thus be written as

(7) \begin{eqnarray*} \phi _1&=|\sigma _1=+1,s_3=+1\rangle , \end{eqnarray*}

(8) \begin{eqnarray*} \phi _2&=|\sigma _1=-1,s_3=-1\rangle . \end{eqnarray*}

The effective Zeeman coupling can be projected onto the subspace of helical edge states Ψ_*p*_(*X*) and the effective Zeeman coupling for helical edge states under an in-plane magnetic field is given by

(9) \begin{eqnarray*} h_{\rm edge}(k_Y)&=&\tilde{A}k_Y \tilde{s}_3+\mu _Bg_{E,X}B_X \tilde{s}_1\nonumber\\ && +\,\,\mu _Bg_{E,Y}B_Y\tilde{s}_2, \end{eqnarray*}

where $\tilde{s}_i$ is the Pauli matrix in helical edge state space and the effective *g*-factors for edge states are *g*_*E*,*X*_ = (*g*_1_ − *g*_2_)/2 and *g*_*E*,*Y*_ = (*g*_1_ + *g*_2_)/2 (more details can be found in the online SM). From the above form of Zeeman coupling, the non-zero values of both *g*_1_ and *g*_2_ make the magnetic gaps of helical edge states different between the parallel and perpendicular magnetic field direction with respect to the edge direction. The anisotropic Zeeman splitting of helical edge states from the aforementioned tight-binding model calculations can be reproduced in the effective model (see the online SM). As the orbital Zeeman term can increase *g*_2_, it will also enhance the anisotropy of Zeeman splitting for edge states. The in-plane Zeeman coupling is isotropic in the bulk but anisotropic at the edges, and such a unique magnetic anisotropy is directly derived from its basis with different total angular momenta $j_z=\pm \frac{1}{2},\pm \frac{3}{2}$ of the BHZ model.

### MZMs at the corner and the edge CPDW

**Figure 3.**
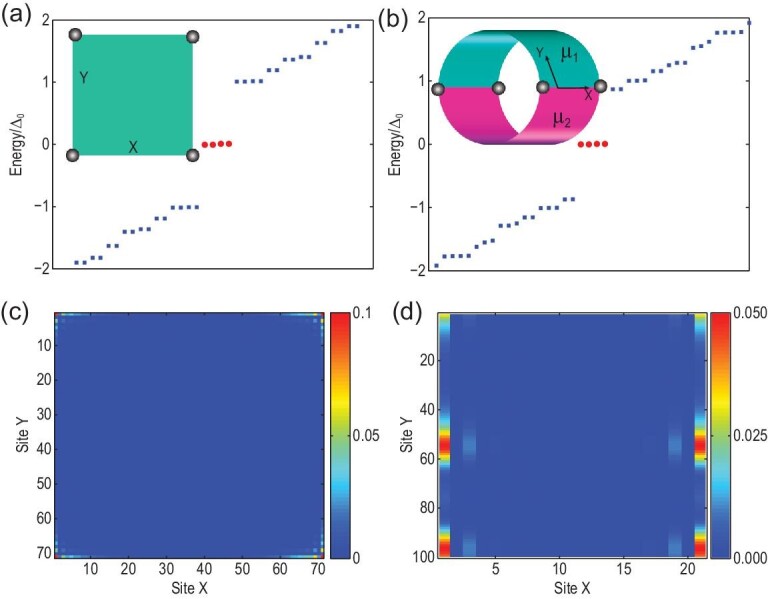
Energy spectra and the spatial pattern for MZMs on the square and CPDW geometries. (a) The energy spectra and (c) the wavefunctions of MZMs are shown for a square geometry with four MZMs labeled by the gray circles in the inset of (a). (b) The energy spectra and (d) the wavefunctions of MZMs are shown for the CPDW geometry with the periodic boundary condition along the *Y* axis and the open boundary condition along the *X* axis in the inset of (d), in which the locations of the four MZMs are also denoted with gray circles. We adopted the parameters given in Section V of the online SM.

Because of the existence of a TPT at the edge, MZMs can appear at the domain wall between phases I and II. To explicitly demonstrate this scenario, we compute its energy levels and show the existence of the MZMs in two different experimental configurations (Fig. 1(b) and (c), as well as the insets in Fig. 3(a) and (b)) based on the effective Hamiltonian *h*_0_ + *h*_*Z*_. In the superconducting phase, the BdG Hamiltonian for the basis $\Psi ^\circ_{\boldsymbol {k}}=(\psi ^\circ_{\boldsymbol {k}},\psi ^T_{-\boldsymbol {k}})$ is given by

(10) \begin{eqnarray*} \mathcal {H}_{\rm BdG}=\sum _{\boldsymbol {k}}\Psi ^\circ_{\boldsymbol {k}}h_{\rm BdG}({\boldsymbol k})\Psi _{\boldsymbol {k}}, \end{eqnarray*}

where

$$\begin{eqnarray*}
&&\!\!\!\!\! h_{\rm BdG}=\nonumber\\
&&\!\!\!\!\! \left(\begin{array}{cc}h_{0}({\boldsymbol k})\!+\! h_Z(-\boldsymbol {k})\!-\!\mu & {\Delta }(\boldsymbol {k}) \\
{\Delta }^\circ(\boldsymbol {k}) & -h^*_{0}(-{\boldsymbol k})\!-\!h^*_Z(-\boldsymbol {k})\!+\!\mu \end{array}\right)
\end{eqnarray*}$$

with ${\Delta }(\boldsymbol {k})=[\Delta _0+\Delta _1-\Delta _1/4(k^2_X+k^2_Y)]s_2\sigma _3$ for extended *s*-wave (*s*_±_) pairing. We note that the superconducting gap is opposite for two orbitals in the BHZ model. Detailed derivations of *h*_0_, *h*_*Z*_ and Δ are provided in the online SM. By choosing appropriate parameters *C*, *D*, *M*, *B*, *A*, *g*_1_, *g*_2_, the effective model can well reproduce the band structure of the tight-binding model near Γ and anisotropic Zeeman splitting (see the online SM). As the topological properties in Fe(Te,Se) monolayers are dominated by the electronic structures around the Γ point, where *s*- and *s*_±_-wave pairings exhibit similar behaviors, Majorana states are robust, irrespective of pairing symmetries in the system.

This model allows us to directly calculate the MZMs in two configurations as depicted in the insets of Fig. 3(a) and (b). We first consider a square geometry with four corners and apply a magnetic field along the *X* direction (θ = 90° relative to the (100) edge). The parameter choice of the BHZ model is discussed and provided in Section V of the online SM, for which we find the (100) edge in phase I and the (010) edge in phase II. As a result, four MZMs appear at zero energy and are well localized at the four corners, as shown in Fig. 3(a) and (c). The appearance of MZMs at the corner also implies that the bulk SC represents a higher-order TSC phase [39–49], for which our system provides a concise experimental platform in an intrinsic and high-*T*_*c*_ SC domain. The localization length of the MZM depends on the velocity of edge states and the superconducting gap. By choosing $v$_*f*_ = 4.4 × 10^3^ m/s and Δ_edge_ = 0.5–1 meV [28], we estimate the localization length as ℏ$v$_*f*_/Δ_edge_ ∼ 30−60 Å, which can be conveniently measured in experiments. Similar calculations can also be performed for the CPDW in a slab configuration with the open boundary condition along the *X* direction and the periodic boundary condition along the *Y* direction, as shown in the inset of Fig. 3(b). By carefully choosing the chemical potentials μ_1_ and μ_2_, the blue and pink regions in the inset of Fig. 3(b) are in phases I and II, respectively, thus allowing for MZMs located at two ends of the CPDW between these two regions. Our calculations in Fig. 3(b) and (d) indeed show four MZMs appearing at zero energy due to two CPDWs in one period of the whole system.

### Electric-field-induced 2D bulk TPT

**Figure 4.**
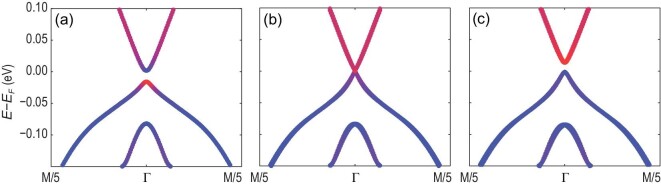
Two-dimensional bulk TPT for the Fe(Te,Se) monolayer as a function of the asymmetric potential induced by the dual gate voltage. The band structure is (a) inverted without electric field, (b) at the critical gapless point at *V*_*g*_ = 0.3 eV and (c) normal at *V*_*g*_ = 0.4 eV. The red represents the *p*_$z$_ orbitals of Te/Se while the blue represents the orbitals from Fe atoms.

Finally, an additional direction to achieve MZMs inside the 2D bulk system is given through patterning local gating to form a tri-junction with three different regions, as labeled by μ_1_, μ_2_ and *V*_*g*3_ in Fig. 1(d). The situation here is quite similar to the edge CPDW in Figs 1(c) and 3(b). We choose the same chemical potentials μ_1_ and μ_2_ for two regions of the tri-junction to be in phases I and II of Fig. 2(d). If we can achieve a trivial insulator phase in the last region *V*_*g*3_ of Fig. 1(d), which is equivalent to the vacuum termination in Fig. 1(c), the tri-junction is topologically equivalent to the edge CPDW and thus allows for the existence of MZMs. Therefore, we next show the existence of a 2D bulk TPT induced by electric gating to tune the 2D Fe(Se,Te) monolayer between a QSH phase and a trivial insulator phase. The key idea here is that the *p*_$z$_ orbital of Se or Te atoms is strongly hybridized with the Fe *d*_*xy*_ orbital, and thus contributes significantly to the odd-parity $j_z=\pm \frac{1}{2}$ bands, but not to the even-parity $j_z=\pm \frac{3}{2}$ bands [25]. Since the Fe layer is sandwiched between two Se (or Te) layers in an Fe(Se,Te) monolayer, the asymmetric potential between two Se (or Te) layers can induce an energy shift of the $j_z=\pm \frac{1}{2}$ bands with respect to the $j_z=\pm \frac{3}{2}$ bands. Thus, if we initially tune the band gap close to zero by controlling the Se/Te composition ratio, the 2D bulk TPT between the QSH state and the trivial insulator can be induced by a dual gate voltage. Our tight-binding model does not explicitly involve the *p*_$z$_ orbital of Se or Te atoms and is thus not ideally suitable for studying this mechanism. Instead, we perform a calculation based on the tight-binding model including both Fe *d* and Se/Te *p* oribtals from the Wannier function method [50] (see the online SM for more technical details). The energy dispersions are shown in Fig. 4(a)–(c) for different asymmetric potentials. The QSH state in Fig. 4(a) and the trivial insulator phase in Fig. 4(c) are separated by a 2D TPT shown in Fig. 4(b). The band inversion can be further revealed by projecting each band to the atomic orbitals. The red and blue colors in Fig. 4(a)–(c) represent the atomic orbital contribution from Fe and Se/Te atoms, from which one can easily see the inverted band structure in Fig. 4(a) and a trivial insulator phase in Fig. 4(c). This band inversion is induced by the inversion symmetry breaking due to the electric gates, as discussed in the online SM. In summary, by making the experimental setup shown in Fig. 1(d), our study indicated that MZMs can indeed be realized in a tri-junction configuration not just limited to corners.

## DISCUSSION

Our work theoretically demonstrates the suitability of a high-*T*_*c*_ SC Fe(Te,Se) monolayer as a platform for the realization of MZMs. This is the first theoretical proposal about the intrinsic material realization of time-reversal-breaking high-order topological superconductivity with Majorana corner modes in iron-based superconductor systems. Moreover, the scenario of edge magnetic anisotropy, even surviving with an *s*-wave pairing, serves as a general principle to search for high-order topological superconductivity in other materials. A magnetic layer, such as CrX_3_ (X = I, Br, Cl) [51], CrGeTe_3_ [52] or FeTe, can be inserted between the Fe(Te,Se) monolayer and the insulator layers in Fig. 1(a) in order to enhance the magnetic gap of helical edge states through the magnetic proximity effect. While the underlying mechanism is similar, this may broaden the parameter regimes for MZMs because of the much stronger exchange interaction compared to the Zeeman coupling. In addition, Josephson junctions may provide an alternative approach to realize MZMs in an Fe(Te,Se) monolayer [53], an approach which has recently been applied to semiconductor/superconductor heterostructures [54,55]. Furthermore, rotating the in-plane magnetic field may provide an efficient approach to perform the braiding operation for the corner MZMs [56]. The 2D nature also makes our platform suitable for the potential manipulation and detection of MZMs, the implementation scheme of which we leave for future work.

## METHODS

We adopted the five-band tight-binding model to investigate the topological phase transition under an in-plane magnetic field with the Hamiltonian elements and corresponding parameters provided in Section I of the online SM. The topological phase transition on edges is directly demonstrated by a topological invariant change with an effective edge Hamiltonian in Section III of the online SM. For Majorana calculations, we adopted the effective model around the Γ point and the parameters are provided in Section V and Table III in the online SM. For the bulk topological phase transition with gating, we construct a tight-binding model with 32 bands, including 20 Fe *d* orbitals and 12 Se *p* orbitals for two sublattices, through the maximum localized Wannier function method, which reproduces well the first principle calculations (see Section VII and Fig. 5 in the online SM).

## Supplementary Material

nwab087_Supplemental_File
