# Supplementary material for: High-Tc superconductor Fe(Se,Te) monolayer: an intrinsic, scalable and electrically tunable Majorana platform
Source: Natl Sci Rev. 2021 May 19;9(3):nwab087. doi: 10.1093/nsr/nwab087 (PMC8924703; doi:10.1093/nsr/nwab087)
Supplement: nwab087_Supplemental_File [file nwab087_supplemental_file.pdf]

Supplementary materials for "High-T_c superconductor Fe(Se,Te) monolayer: a Scalable Electrically-tunable Majorana Platform"

Xianxin Wu,[1] Xin Liu,[2,3] Ronny Thomale,[1] and Chao-Xing Liu[4,*]

[1]Institut für Theoretische Physik und Astrophysik, Julius-Maximilians-Universität Würzburg, 97074 Würzburg, Germany
[2]School of Physics, Huazhong University of Science and Technology, Wuhan, Hubei 430074, China
[3]Wuhan National High Magnetic Field Center, Huazhong University of Science and Technology, Wuhan, Hubei 430074, China
[4]Department of Physics, the Pennsylvania State University, University Park, PA, 16802

I. PARAMETERS IN THE TIGHT-BINDING MODEL AND SPIN-ORBIT COUPLING MATRIX IN FE(TE,SE)

The tight-binding model are written in the basis of five Fe d orbitals, namely $d_{xz}, d_{yz}, d_{x^2-y^2}, d_{xy}, d_{z^2}$ and its form can be found in Ref.1 and Ref.2. The on-site energies and hopping parameters for the tight-binding model can be found in Table I. In addition, we include the on-site spin-orbit coupling matrix for d orbitals, given by

$$M = \begin{pmatrix} M^{\uparrow\uparrow} & M^{\uparrow\downarrow} \\ M^{\downarrow\uparrow} & M^{\downarrow\downarrow} \end{pmatrix}, \tag{1}$$

$$M^{\uparrow\uparrow/\downarrow\downarrow} = \pm\lambda_{soc} \begin{pmatrix} 0 & -\frac{i}{2} & 0 & 0 & 0 \\ \frac{i}{2} & 0 & 0 & 0 & 0 \\ 0 & 0 & 0 & -i & 0 \\ 0 & 0 & i & 0 & 0 \\ 0 & 0 & 0 & 0 & 0 \end{pmatrix}, \tag{2}$$

$$M^{\uparrow\downarrow} = \lambda_{soc} \begin{pmatrix} 0 & 0 & -\frac{1}{2} & \frac{i}{2} & \frac{\sqrt{3}}{2} \\ 0 & 0 & -\frac{i}{2} & -\frac{1}{2} & -\frac{i\sqrt{3}}{2} \\ \frac{1}{2} & \frac{i}{2} & 0 & 0 & 0 \\ -\frac{i}{2} & \frac{1}{2} & 0 & 0 & 0 \\ -\frac{\sqrt{3}}{2} & \frac{i\sqrt{3}}{2} & 0 & 0 & 0 \end{pmatrix}, \tag{3}$$

$$M^{\downarrow\uparrow} = [M^{\uparrow\downarrow}]^{\dagger}, \tag{4}$$

with the spin-orbit coupling parameter $\lambda_{so} = 80$ meV for the tight-binding model.

TABLE I: Hopping parameters for monolayer Fe(Te,Se) in the tight binding model. The x direction is along the Fe-Fe bond. The onsite energies of d oribtals are (all in eV) : ϵ_1=0.1337, ϵ_3=-0.3993, ϵ_4=-0.1513, ϵ_5=-0.3193 and $\mu = 0$.

t_i^{mn}	i=x	i=y	i=xy	i=xx	i=yy	i=xxy	i=xyy	i=$xxyy$
mn=11	-0.1444	-0.4009	0.227	0.002	-0.036	-0.019	0.014	0.024
mn=33	0.4584		-0.070	-0.013				0.012
mn=44	0.0604		0.0200	0.002		-0.019		-0.024
mn=55			0.013	-0.014		0.006		-0.011
mn=12			0.103			-0.011		0.032
mn=13		0.473	-0.089		0.011	-0.018	0.006	
mn=14	0.2736		0.053	-0.001		0.006		-0.009
mn=15		0.2	-0.13		0.009	-0.009	-0.011	-0.012
mn=34						0.012		
mn=35	-0.401			-0.023		-0.006		
mn=45			-0.113					0.011

TABLE II: Parameters in the BHZ model for monolayer Fe(Te,Se), fitting with band structures from tight binding model.

C(eV)	M(eV)	D(eVÅ^2)	B(eVÅ^2)	A(eVÅ)
-0.0227	-0.0538	3.3	-4.1	1.8

A. Angular momentum operators and Zeeman coupling in d orbital space

The angular momentum operators in d orbital space are given by,

$$L_x = \begin{pmatrix} 0 & 0 & 0 & i & 0 \\ 0 & 0 & -i & 0 & -\sqrt{3}i \\ 0 & i & 0 & 0 & 0 \\ -i & 0 & 0 & 0 & 0 \\ 0 & \sqrt{3}i & 0 & 0 & 0 \end{pmatrix}, \tag{5}$$

$$L_y = \begin{pmatrix} 0 & 0 & -i & 0 & \sqrt{3}i \\ 0 & 0 & 0 & -i & 0 \\ i & 0 & 0 & 0 & 0 \\ 0 & i & 0 & 0 & 0 \\ -\sqrt{3}i & 0 & 0 & 0 & 0 \end{pmatrix}, \tag{6}$$

$$L_z = \begin{pmatrix} 0 & -i & 0 & 0 & 0 \\ i & 0 & 0 & 0 & 0 \\ 0 & 0 & 0 & -2i & 0 \\ 0 & 0 & 2i & 0 & 0 \\ 0 & 0 & 0 & 0 & 0 \end{pmatrix}, \tag{7}$$

where the basis order is xz, yz, x^2-y^2, xy, z^2. The Zeeman coupling in the tight binding model reads

$$\mathcal{H}_Z = \boldsymbol{\mu} \cdot \boldsymbol{B} = \mu_B(g_s \boldsymbol{S} + g_l \boldsymbol{L}) \cdot \boldsymbol{B}, \tag{8}$$

in which the first and the second terms describe spin and orbital Zeeman coupling, $\boldsymbol{s} = \frac{1}{2}\boldsymbol{\sigma}$, $g_s = g_0$ and $g_l = g_0/2$.

II. EFFECTIVE MODELS AROUND Γ POINT AND ANISOTROPIC ZEEMAN COUPLING FOR HELICAL EDGE STATES

In the main text, we study the topological phase transition at the 1D edge and the phase diagram based on a realistic microscopic tight-binding model with 20 bands for Fe(Te,Se) monolayer. This model can reproduce well most of the details of the electronic band structures in the whole Brillouin zone from the first principles calculations, and thus makes our calculations realistic. However, for the calculation of the Majorana corner states, we need to consider a finite sample with open boundary conditions in all directions, and using a realistic 20-band model for the calculation in a finite sample is unrealistic. Thus, we will develop an effective model to capture the main physics around Γ point first and then apply it to the study of the corner Majorana modes.

A. Bases and Band structure of the BHZ model

From the tight-binding model, we identify the band inversion at Γ occurring between the odd-parity $j_z = \pm\frac{1}{2}$ and even-parity $j_z = \pm\frac{3}{2}$ bands, which form the bases of the effective model, the Bernevig-Hughes-Zhang (BHZ) model, as described by the Eq. (4) in the main text. The corresponding wavefunctions read,

$$|\Gamma_6^-, +\frac{1}{2}\rangle = +v_1|d_{xy}^-, \uparrow\rangle + v_2|d_{x^2}^-, \uparrow\rangle + v_3(-i|d_{yz}^-, \downarrow\rangle + |d_{xz}^-, \downarrow\rangle) \tag{9}$$

$$|\Gamma_6^-, -\frac{1}{2}\rangle = +v_1^*|d_{xy}^-, \downarrow\rangle + v_2^*|d_{x^2}^-, \downarrow\rangle - v_3^*(i|d_{yz}^-, \uparrow\rangle + |d_{xz}^-, \uparrow\rangle) \tag{10}$$

$$|\Gamma_7^+, +\frac{3}{2}\rangle = -w_1(i|d_{yz}^+, \uparrow\rangle + |d_{xz}^+, \uparrow\rangle) + w_2|d_{xy}^+, \downarrow\rangle + w_3|d_{x^2}^+, \downarrow\rangle \tag{11}$$

$$|\Gamma_7^+, -\frac{3}{2}\rangle = +w_1^*(-i|d_{yz}^+, \downarrow\rangle + |d_{xz}^+, \downarrow\rangle) + w_2^*|d_{xy}^+, \uparrow\rangle + w_3^*|d_{x^2}^+, \uparrow\rangle, \tag{12}$$

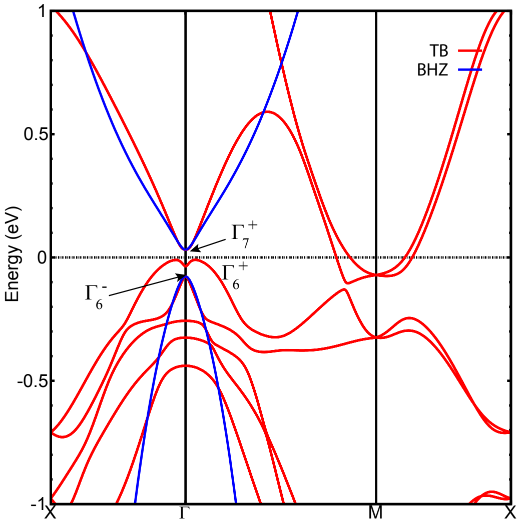

FIG. 1: (color online) Band structures from tight binding model(red) and BHZ model(blue). Here $+/-$ denotes the parity-even/parity-odd state.

where $|X_m^{\pm}\rangle = (|X_m^A\rangle \pm |X_m^B\rangle)$ with the sublattices A, B and $X_m = d_{xz}, d_{yz}, d_{x^2}, d_{xy}$. Here x/y is along Fe-Fe direction (see Fig.1(e) in the main text). The $|\Gamma_6^-, \pm\frac{1}{2}\rangle$ bands are dominated by the d_{xy} orbitals ($|v_1| \gg |v_2|, |v_3|$) while the $|\Gamma_7^+, \pm\frac{3}{2}\rangle$ bands are mainly contributed from the $d_{xz,yz}$ and d_{xy} orbitals ($|w_1|, |w_2| \gg |w_3|$). All the parameters in the above wavefunctions can be obtained by diagonalizing the tight-binding model numerically, from which we find the eigenstates at Γ point are (with a small exchange field to split two fold degeneracy)

$$|\Gamma_6^-, +\frac{1}{2}\rangle = -(0.70 + 0.03i)|d_{xy}^-, \uparrow\rangle - 0.05i|d_{x^2}^-, \uparrow\rangle + 0.01i(-i|d_{yz}^-, \downarrow\rangle + |d_{xz}^-, \downarrow\rangle) \quad (13)$$

$$|\Gamma_6^-, -\frac{1}{2}\rangle = -(0.70 - 0.03i)|d_{xy}^-, \downarrow\rangle - 0.05i|d_{x^2}^-, \downarrow\rangle + 0.01i(i|d_{yz}^-, \uparrow\rangle + |d_{xz}^-, \uparrow\rangle) \quad (14)$$

$$|\Gamma_7^+, +\frac{3}{2}\rangle = -(0.36 + 0.34i)(i|d_{yz}^+, \uparrow\rangle + |d_{xz}^+, \uparrow\rangle) - (0.1 - 0.1i)|d_{xy}^+, \downarrow\rangle) + (0.01 + 0.01i)|d_{x^2}^+, \downarrow\rangle) \quad (15)$$

$$|\Gamma_7^+, -\frac{3}{2}\rangle = (0.36 - 0.34i)(-i|d_{yz}^+, \downarrow\rangle + |d_{xz}^+, \downarrow\rangle) - (0.1 + 0.1i)|d_{xy}^+, \uparrow\rangle) + (0.01 - 0.01i)|d_{x^2}^+, \uparrow\rangle). \quad (16)$$

The parameters of the BHZ model (Eq. (4) in the main text) are given in the Table II, and we calculate the band structure of the BHZ model, as shown by the blue lines in Fig.1, which fits well with the relevant bands around Γ point obtained from the tight-binding model (red lines in Fig.1). It should be pointed out that the highest energy valence band comes from the even-parity $j_z = \pm\frac{1}{2}$ state. Since this band share the same parity as the conduction band (even-parity $j_z = \pm\frac{3}{2}$ band), it is thus not important for topological properties in Fe(Te,Se)[1]. Hence, we omit this band in the effective model.

B. Zeeman coupling in the BHZ model

Based on the above form of the wavefunctions at Γ, we can project the Zeeman coupling (the Hamiltonian $\mathcal{H}_Z$ in Eq. (3) of the main text) into the low energy subspace of the BHZ model and the effective Zeeman coupling term is given by

$$\tilde{h}_Z = g_0\mu_B \begin{pmatrix} 2|v_1|^2 B_z & 0 & 2v_1^{*2}(B_x - iB_y) & 0 \\ 0 & (4|w_1|^2 - 2|w_2|^2)B_z & 0 & (2w_2^{*2} - 4iw_1^* w_2^* \frac{g_l}{g_0})(B_x + iB_y) \\ 2v_1^2(B_x + iB_y) & 0 & -2|v_1|^2 B_z & 0 \\ 0 & (2w_2^2 - 4iw_1 w_2 \frac{g_l}{g_0})(B_x - iB_y) & 0 & -(4|w_1|^2 - 2|w_2|^2)B_z \end{pmatrix} \quad (17)$$

in the $x - y$ coordinate system. Here v_1^2 and w_2^2 coefficients are from the spin Zeeman coupling while $w_1 w_2$ coefficient is from the orbital Zeeman coupling in Eq.8. Though a unitary transformation, the Zeeman coupling in the $X - Y$ coordinate system

reads,

$$h_Z = \mu_B \begin{pmatrix} g_1^{\perp} B_Z & 0 & g_1^{\|}(B_X - iB_Y) & 0 \\ 0 & g_2^{\perp} B_Z & 0 & g_2^{\|}(B_X + iB_Y) \\ g_1^{\|}(B_X + iB_Y) & 0 & -g_1^{\perp} B_Z & 0 \\ 0 & g_2^{\|}(B_X - iB_Y) & 0 & -g_2^{\perp} B_Z \end{pmatrix} \tag{18}$$

$$= \mu_B(g_1^{\perp} P_{1/2} + g_2^{\perp} P_{3/2}) B_Z s_3 + \mu_B(g_1^{\|} P_{1/2} + g_2^{\|} P_{3/2}) B_X s_1 + \mu_B(g_1^{\|} P_{1/2} - g_2^{\|} P_{3/2}) B_Y s_2, \tag{19}$$

$$g_1^{\perp} = 2g_0|v_1|^2, \quad g_2^{\perp} = g_0(4|w_1|^2 - 2|w_2|^2), \tag{20}$$

$$g_1^{\|} = g_0|\frac{1}{\sqrt{2}} v_1^{*2}(1-i)|, \quad g_2^{\|} = -g_0|\frac{1}{\sqrt{2}}(w_2^{*2} - i w_1^* w_2^*)(1+i)|. \tag{21}$$

Here $g_l = \frac{g_0}{2}$, $P_{1/2(3/2)} = (\sigma_0 + (-)\sigma_3)/2$ is the projector operator in the subspace of the $j_z = \pm 1/2 (\pm 3/2)$ states and $g_{1,2}^{\perp,\|}$ are the effective g-factors depending on material details. The orbital Zeeman term can greatly enhance $g_2^{\|}$ due to $|w_1| > |w_2|$.

C. Effective pairing in the BHZ model

In the main text, we consider the intraorbital pairing on the same sublattice. Now we derive the pairing terms in the bases of BHZ model. From Eq.12, we find that the s-wave even-parity pairing cannot couple the $j_z = \pm\frac{1}{2}$ and $j_z = \pm\frac{3}{2}$ states due to their opposite parities under inversion symmetry. After omitting the $\boldsymbol{k}$ dependent part of wavefunctions, the intraorbital pairing is given by

$$\langle c^{\dagger}_{\boldsymbol{k},\frac{1}{2}} c^{\dagger}_{-\boldsymbol{k},-\frac{1}{2}} \rangle = 2|v_1|^2 \Delta_{xy}, \tag{22}$$

$$\langle c^{\dagger}_{\boldsymbol{k},\frac{3}{2}} c^{\dagger}_{-\boldsymbol{k},-\frac{3}{2}} \rangle = -4|w_1|^2 \Delta_{xz/yz} - 2|w_1|^2 \Delta_{xy}. \tag{23}$$

We notice that the pairing gaps are opposite for $j_z = \pm\frac{1}{2}$ and $j_z = \pm\frac{3}{2}$ states, generating the adopted pairing $\Delta(\boldsymbol{k}) = \Delta_k s_2 \sigma_3$ in BHZ model in the main text.

D. Anisotropic Zeeman coupling for helical edge states

To investigate the effective Zeeman coupling of helical edge states, we first calculate the helical edge state from the BHZ Hamiltonian. For the (100) edge, we can replace $k_X \to -i\partial_X$ and omit the $\epsilon_0(\boldsymbol{k})$ term. The Hamiltonian reads

$$h_0(-i\partial_X, k_Y) = [M - B(-\partial_X^2 + k_Y^2)]\sigma_3 + A(k_Y s_0 \sigma_1 - i\partial_X s_3 \sigma_2), \tag{24}$$

of which the eigenvalue equations $h_0 \Psi_p(X) = E_p \Psi_p(X)$ at $k_Y = 0$ under the boundary condition $\Psi_p(X \to 0) = \Psi_p(X \to +\infty) = 0$ can be solved and two zero-energy mode solutions are given by

$$\Psi_p(X) = N sinh(\eta_1 X) e^{\eta_2 X} \phi_p, \tag{25}$$

where the normalization factor $N = 4|\eta_2(\eta_2^2 - \eta_1^2)/\eta_1^2|$, $\eta_1 = \sqrt{\frac{A^2}{4B^2} - \frac{M}{B}}$ and $\eta_2 = \frac{A}{2B}$. The eigenvectors ϕ_p satisfy the eigenequation $s_3 \sigma_1 \phi_p = \phi_p$ and thus can be written as

$$\phi_1 = |\sigma_1 = +1, s_3 = +1\rangle, \tag{26}$$

$$\phi_2 = |\sigma_1 = -1, s_3 = -1\rangle. \tag{27}$$

The effective Zeeman coupling can be projected into the subspace of helical edge states $\Psi_p(X)$ and its matrix elements read

$$\langle \phi_1 | H_z | \phi_1 \rangle = \mu_B (g_1^{\perp} + g_2^{\perp}) B_Z / 2, \tag{28}$$

$$\langle \phi_1 | H_z | \phi_2 \rangle = \mu_B (g_1^{\|} - g_2^{\|}) B_X / 2 - i\mu_B (g_1^{\|} + g_2^{\|}) B_Y / 2. \tag{29}$$

Thus, the effective Zeeman coupling for helical edge states under a magnetic field is given by

$$h_{edge}(k_Y) = \begin{pmatrix} \tilde{A} k_Y + \mu_B g_{E,Z}^{\perp} B_Z & \mu_B g_{E,X}^{\|} B_X - i\mu_B g_{E,Y}^{\|} B_Y \\ \mu_B g_{E,X}^{\|} B_X + i\mu_B g_{E,Y}^{\|} B_Y & -\tilde{A} k_Y - \mu_B g_{E,Z}^{\perp} B_Z \end{pmatrix},$$

$$= (\tilde{A} k_Y + \mu_B g_{E,Z}^{\perp} B_Z)\tilde{s}_3 + \mu_B g_{E,X}^{\|} B_X \tilde{s}_1 + \mu_B g_{E,Y}^{\|} B_Y \tilde{s}_2, \tag{30}$$

with $g^{\perp}_{E,Z} = (g^{\perp}_1 + g^{\perp}_2)/2$, $g^{\parallel}_{E,X} = (g^{\parallel}_1 - g^{\parallel}_2)/2$ and $g^{\parallel}_{E,Y} = (g^{\parallel}_1 + g^{\parallel}_2)/2$. From the above form of Zeeman coupling, the non-zero values of both $g^{\parallel}_1$ and $g^{\parallel}_2$ make the magnetic gaps of helical edge states different between the parallel and perpendicular magnetic field direction with respect to the edge direction, which is consistent with the anisotropic Zeeman splitting of helical edge states from tight binding model calculations. This also indicates that the magnetic gaps of helical edge states are different at two orthogonal edges when the magnetic field is along one of the edges. As the orbital Zeeman term can enhance $g^{\parallel}_2$, it will also enhance the anisotropy of Zeeman splitting for edge states. This magnetic anisotropy is essential for the existence of Majorana zero modes at the corner.

III. TOPOLOGICAL PHASE TRANSITION ON THE EDGE

A. 1D effective model

The effective edge Hamiltonian described by,

$$H_{TI} = \sum_{\mathbf{k}} \psi^{\dagger}(\mathbf{k})(vk\tilde{s}_3 - \mu)\psi(\mathbf{k}) \tag{31}$$

where v is the edge-state velocity, μ is the chemical potential and $\psi^{\dagger}(\mathbf{k}) = [\phi^{\dagger}_1(\mathbf{k}), \phi^{\dagger}_2(\mathbf{k})]$. Below T$_c$, the Fe(Te,Se) monolayer become superconducting. We consider an s-wave spin singlet pairing in Fe(Te,Se), the superconducting proximity effect on the edge can be modeled with a Hamiltonian,

$$H_S = \sum_{\mathbf{k}} \Delta\psi_{\downarrow}(-\mathbf{k})\psi_{\uparrow}(\mathbf{k}) + h.c.. \tag{32}$$

Moreover, we can introduce an inplane Zeeman field,

$$H_Z = -h\sum_{\mathbf{k}} \psi^{\dagger}(\mathbf{k})\tilde{s}_1\psi(\mathbf{k}). \tag{33}$$

Therefore, the full Hamiltonian for the edge states is given by,

$$H_{edge} = H_{TI} + H_Z + H_S = \sum_{\boldsymbol{k}} \Psi^{\dagger}(\boldsymbol{k})H_k\Psi(\boldsymbol{k}), \tag{34}$$

$$H_k = vk\tilde{\tau}_0\tilde{s}_3 - \mu\tilde{\tau}_3\tilde{s}_0 + h\tilde{\tau}_3\tilde{s}_1 - \Delta\tilde{\tau}_2\tilde{s}_2, \tag{35}$$

where $\tilde{s}$ and $\tilde{\tau}$ label the Pauli matrices in spin and Nambu space. Specifically, when $\Delta = 0$, the energy spectrum is $\epsilon_{\pm}(k) = -\mu \pm \sqrt{(vk)^2 + h^2}$ and $\psi_{k\pm}$ create electrons with energy $\epsilon_{\pm}(k)$ on the edge. The linear bands become two quadratic bands with a gap at $k = 0$. In terms of the operators $\psi_{k\pm}$, the singlet pairing term H_S can be written as,

$$\begin{aligned} H_S &= \sum_{\mathbf{k}}[\frac{\Delta_p(\mathbf{k})}{2}(\psi_{-\mathbf{k}+}\psi_{\mathbf{k}+} + \psi_{-\mathbf{k}-}\psi_{\mathbf{k}-} + h.c.) \\ &\quad +\Delta_s(\mathbf{k})(\psi_{-\mathbf{k}+}\psi_{\mathbf{k}-} + h.c.)] \end{aligned} \tag{36}$$

where $\Delta_p(\mathbf{k}) = -\frac{vk\Delta}{\sqrt{v^2k^2+h^2}}$ and $\Delta_s(\mathbf{k}) = -\frac{h\Delta}{\sqrt{v^2k^2+h^2}}$. Therefore, effectively the model describes a system with interband s-wave pairing and intraband p-wave pairing. If the Fermi level just crosses one edge band, we can omit the other band and the system is 1D p-wave superconducting[3]. If the chemical potential only crosses the upper part of the bands, the lower band plays no roles and can be projected out by sending $\psi_{\pm\mathbf{k}-} \to 0$. With $h \gg \Delta$, $\epsilon_{+}(k) \approx (h-\mu) + \frac{v^2}{2h}k^2 = \mu_{eff} + k^2/2m_{eff}$ around $k = 0$ point and $\Delta_p(\mathbf{k}) \approx -\frac{v\Delta}{h}k = \Delta_{eff}k$. Now the effective Hamiltonian matrix in momentum space can be written as $H_{eff}(k) = [-(\mu - h) + \frac{v^2}{2h}k^2]\bar{\tau}_3 - \frac{v\Delta}{h}k\bar{\tau}_1$, where $\bar{\boldsymbol{\tau}}$ label the Pauli matrices in Nambu space for the upper band ψ_{+}. This Hamiltonian in real space can be further written as,

$$H_{eff} = \int dx[\psi^{\dagger}_{+}(-\frac{\partial^2_x}{2m_{eff}} - \mu_{eff})\psi_{+} + \frac{\Delta_{eff}}{2}(-\psi_{+}i\partial_x\psi_{+} + h.c.)], \tag{37}$$

which describes Kitaev's model for a 1D spinless p-wave superconductor. In the 1D spinless p-wave Kitaev's model, the model belongs to the D class and thus a Z_2 topological invariant can be defined. The system is in the weak-pairing (strong) regime for $|\mu_{eff}| < t_{eff}$ ($|\mu_{eff}| > t_{eff}$) hence topologically nontrivial $Z_2 = 1$ (trivial $Z_2 = 0$)[3]. With $h \gg \Delta$, the system is a topologically trivial phase for $|\mu| < h$ and a topologically nontrivial phase for $|\mu| > h$. The accurate topological criterion is $h < \sqrt{\mu^2 + \Delta^2}$, where the superconducting gap dominates the magnetic gap. For $h > \sqrt{\mu^2 + \Delta^2}$, the magnetic gap dominates the superconducting gap and the system is in a topologically nontrivial phase. In our calculations with increasing magnetic field, the gap first closes and then reopens, which is a clear evidence for topological phase transition (from a Z_2 nontrial to Z_2 trivial).

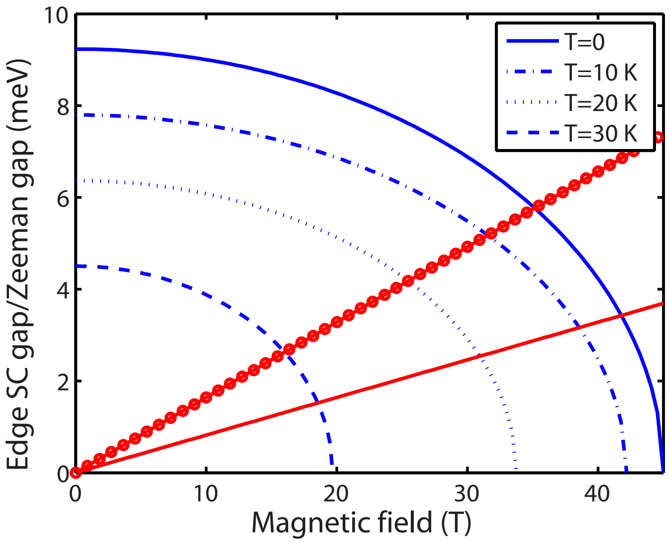

FIG. 2: (color online) Edge superconducting gap (blue lines) and Zeeman splitting (red lines) as a function of the magnetic field at different temperatures. The circle dotted line denotes the Zeeman splitting with a double g factor.

B. Critical magnetic field with a realistic superconducting gap

In the main text, the adopted superconducting gap is 1.3 meV. For monolayer Fe(Te,Se), however, the superconducting gap varies from 12 meV to 16 meV[4]. According to ARPES measurements, a topological phase transition occurs in Fe($Te_{1-x}Se_x$) monolayer only when $x < 0.21$. The corresponding bulk superconducting gap is about 12 meV[4]. Although this gap is much larger than the value adopted in the main text, a magnetic-field driven topological phase transition will always occur if we consider the temperature and magnetic field dependence of the superconducting gap. We performed such an estimation of the critical field by including the B and T dependence of the superconducting gap $\Delta_1(B,T) = \Delta_1(T)\sqrt{1 - B^2/B_c^2(T)}$ at a fixed temperature T, where we used $\Delta_1(T) = \Delta_1(0)\sqrt{1 - T/T_c}$ and $B_c(T) = B_c(0)(1 - T^2/T_c^2)$ in the BCS theory. The adopted parameters are $\Delta_1(0) = 12$ meV, $B_c(0) = 45$ T and $T_c = 40$ K. Fig.2 displays the edge superconducting gap and Zeeman splitting as a function of the magnetic field at different temperatures. The red solid line denotes the Zeeman splitting using the effective g factor in the TB model. The crossing points between the red lines and blue lines are just the phase transition points. We can find that there is always a topological phase transition at different temperature. Moreover, the critical field will get reduced when increasing temperature, suggesting that Fe(Te,Se) is a ideal high-temperature platform. As the TB model can only qualitatively describe Fe(Te,Se) systems, the estimated effective g factor can be inaccurate. If we double the g factor on edges, we get the Zeeman splitting in the circle dotted line and the corresponding critical field is reduced. Therefore, it is promising that the topological transition can be realized in experiments.

IV. TOPOLOGICAL INVARIANT FOR THE APPEARANCE OF MZM

For the appearance of Majorana states, we define a topological invariant from the edge states. For the helical edge states from 2D TI coupled with an s wave superconductor, the superconducting gap is isotropic and thus is the same for the (100) and (010) edges, denoted as X and Y edges below. On the other hand, in-plane magnetic field can induce the Zeeman splitting of helical edge states, which is anisotropic for different edges. Thus, we can label the Zeeman term by $M_i\tilde{\tau}_3\tilde{s}_1$ with $i = X, Y$ for two perpendicular edges. Since the Zeeman term and the superconducting gap term commute with each other, the overall gap of the helical edge states is given by $\Delta_s \pm M_i$ with $i = X, Y$. The topological invariant can be constructed by $\nu = \prod_{i=1}^{2} \nu_{iX}\nu_{iY}$, where $i = 1, 2$ labels two negative energy eigen states, and ν_{iX} and ν_{iY} label the eigenvalues of the operator $\Delta_s\tilde{\tau}_2\tilde{s}_2$ (which commutes with the whole effective edge Hamiltonian) for the corresponding states at $k = 0$ point. By tuning magnetic fields, when $M_Y < \Delta_s < M_X$ (assume $M_Y < M_X$ here), $\nu_{1X} = -\nu_{2X} = -1$ and $\nu_{1Y} = \nu_{2Y} = -1$, and thus $\nu = -1$, corresponding to the existence of one isolated MZM at each corner (topologically nontrivial case). On the other hand, when $\Delta_s < M_X, M_Y$ or $\Delta_s > M_X, M_Y$, we have either $\nu_{1X} = -\nu_{2X} = \nu_{1X} = -\nu_{2Y} = -1$ or $\nu_{1X} = \nu_{2X} = \nu_{1X} = \nu_{2Y} = -1$, and in both cases, $\nu = 1$ and thus the whole system is topologically trivial without any MZM at the corner. The above definition of Z_2 topological invariant is equivalent to that of the Kitaevs model[3].

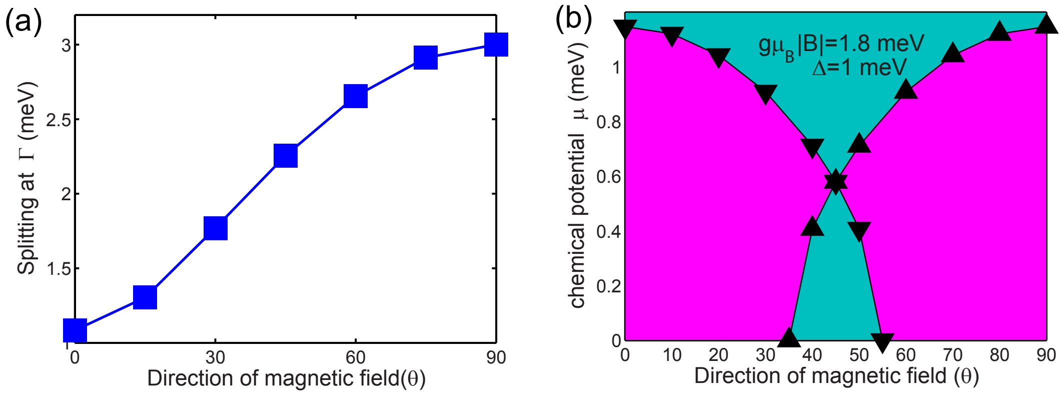

FIG. 3: (color online) (a) Zeeman splitting of edge states as a function of magnetic field angle with respect to 1D edge (Y axis), where $|g_0\mu_B B|$ is fixed to be 1.8 meV. (b) Phase diagram of corner Majorana states as function of chemical potential μ and magnetic field direction. The two edge states have different (pink)/ the same(blue) topological characters, thus can/cannot host corner Majorana states. All calculations are done with the effective model.

TABLE III: Adopted parameters in BHZ model to calculate the anisotropic splitting and phase diagram in Fig.3. In order to calculate the edge states, the BHZ model is rewritten on a square lattice and a lattice constant $a_0 = 12$ Å is adopted.

C(eV)	M(eV)	D(eV Å^2)	B(eV Å^2)	A(eV Å)	$g_1^{\parallel}$	$g_2^{\parallel}$	Δ_0 (meV)
0	-0.0538	0	-4.1	1.8	-0.275g_0	0.55g_0	1.0

V. PARAMETERS IN THE CALCULATIONS OF MAJORANA ZERO MODES

To reproduce the anisotropic splitting in the tight binding model, we use the effective Hamiltonian $H_{eff}(\boldsymbol{k}) = h_0(\boldsymbol{k}) + h_Z$, where h_Z is defined in Eq.19. By adopting the parameters listed in Table III (we have neglected $\epsilon(\boldsymbol{k})$ term and the other parameters are the same as Table II), we obtained the anisotropic splitting for edge states and the phase diagram of corner Majorana states as function of chemical potential μ and magnetic field direction, as shown in Fig.3, similar to the results from tight-binding model (Fig.2 in the main text). It indicates that the BHZ model captures the main physics.

However, the above parameters are not convenient for the direct calculations of Majorana zero modes since a large enough sample size is required, which is beyond our calculation capability. To minimize this finite size effect, we adopted the parameters given in Table III but set $g_1^{\parallel} = -0.55g_0$ to enhance the anisotropy of Zeeman coupling. In the calculation of Majorana corner states (71 × 71 lattices are used), the magnetic field is set to be $g_0\mu_B B_X = 18.2$ meV, μ is set to be zero (the blue star in Fig.4) and the superconducting gap is set to be 10 meV, thus the (100) edge states is in the phase I, as shown in Fig.4. On the other hand, it turns out that the (010) edge is always in the phase II for the whole parameter regime of the $B - \mu$ phase diagram. Therefore, these results suggest the appearance of Majorana corner states (Fig.3(c) in the main text). For the calculations of chemical potential domain wall, we adopted $\mu_1 = 0$ and $\mu_2 = 0.03$ eV with $g_0\mu_B B_X = 18.2$ meV and a slab configuration with the open boundary condition along the X direction and the periodic boundary condition along Y, where the blue star and square represent two parameters in Fig.4. As the two regions have distinct topological properties (See Fig.4), four Majorana states will appear the domain boundaries, as shown in Fig.3(d) in the main text.

VI. DISORDER EFFECT ON THE MZMS

The Majorana states has a topological origin thus are insensitive to weak disorders in the bulk and edge. To demonstrate it, we perform calculations with potential disorder and magnetic disorder. For a uniform-distributed chemical potential disorder in $[-W, W]$ with $W = 0.1\Delta_0$, the energy spectra and wavefunctions of MZMs are displayed in Fig.5(a). We find that only bulk energies are randomly shifted and MZMs are robust against potential disorder. Fig.5(b) show the energy spectra and wavefunctions of MZM with a uniformed-distributed magnetic disorder in $[-M_x, M_x]$ ($M_x = 0.1g\mu_B|B| = 1.82$ meV). These disorder has on effect on the energies of MZMs. Providing the disorder doesnot destroy the bulk superconducting gap, MZMs are shown to be robust according to our calculations. Therefore, the obtained MZMs are robust against both disorder and edge roughness, which provides additional supports for the topological nature of MZMs.

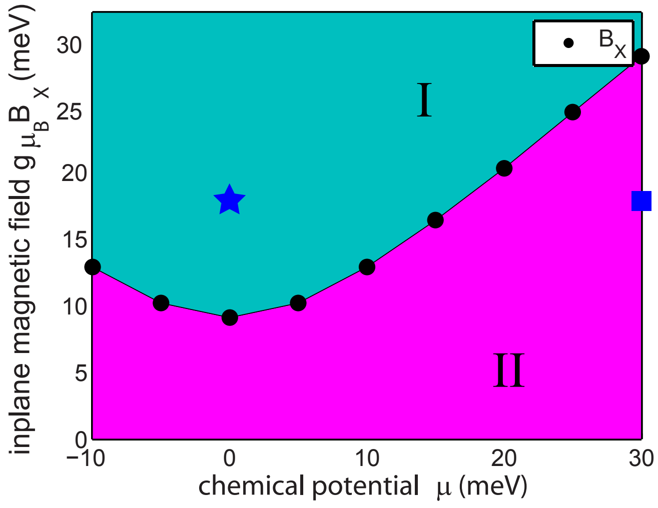

FIG. 4: (color online) TPT as a function of magnetic fields and chemical potentials for the (100) edge by using the BHZ model with parameters listed in Table III (here we used $g_1^{||} = -0.55g_0$ to enhance the anisotropy of Zeeman splitting). The black circles correspond to the TPT line for the magnetic field B_X. For magnetic field along Y, the system is in the phase II in the above parameter region. Here the pairing gap is fixed to be $\Delta_0 = 0.01$ eV.

VII. BULK TOPOLOGICAL PHASE TRANSITION WITH GATING

From the Ref.[33], we find that the odd-parity $j_z = \pm\frac{1}{2}$ state is dominantly contributed by Se/Te p_z orbital and Fe d_{xy} orbital while even-parity $j_z = \pm\frac{3}{2}$ state is mainly attributed to Fe $d_{xz/yz}$ orbitals. It turns out that the distance between Se/Te layer and Fe layer can be tuned by the Se/Te composition, namely a larger Te ratio increasing Se/Te layer height. As a result, increasing the Se/Te height to the Fe layer can weaken the coupling between p_z and d_{xy} orbital, which can shift $j_z = \pm\frac{1}{2}$ state towards lower energy and induce band inversion, driving the system into a topologically nontrivial phase. In the simulation, it is difficult to directly consider the Te/Se substitution. Instead, we can tune the anion height to the Fe layer to mimic the substitution process. Indeed, the inverted band structure can be achieved by choosing the lattice constant $a = 3.905$ Å and anion height $h = 1.52$ Å for monolayer FeSe. We construct a tight-binding model with 32 bands, including 20 Fe d orbitals and 12 Se p orbitals for two sublattices, through the maximum localized Wannier function method, which reproduces well the first principles calculations. The obtained band structure matches well with that from DFT, as shown in Fig.6.

As illustrated in the main text, since two Se/Te layers are distributed on two sides of the Fe layer, it is also possible to induce the band inversion by an asymmetric potential generated by the dual electric gate. The asymmetrical potential in experiments is modelled as

$$\mathcal{H}_P = V_g \sum_{im'\sigma} [c^\dagger_{Am'\sigma}(i)c_{Am'\sigma}(i) - c^\dagger_{Bm'\sigma}(i)c_{Bm'\sigma}(i)], \quad (38)$$

where A and B labels two Se/Te atoms in one unit cell (one above and one below the Fe layers) and $m' = p_x, p_y, p_z$ is the orbital for Te/Se atoms. This asymmetric potential can be added into the 32-band tight-binding model phenomenologically and from Fig. 4 in the main text, we indeed find that the asymmetric potential can shift the valence bands of $j_z = \pm\frac{1}{2}$ odd-parity state and induce topological phase transition. Physically, the influence of asymmetric potential on the $j_z = \pm\frac{1}{2}$ bands can be understood through the second order perturbation theory. As discussed above, the p_z orbital of Te/Se atoms contribute significantly to the $j_z = \pm\frac{1}{2}$ bands at Γ. Since there are two Te/Se atoms in one unit cell (A,B sublattices), we may consider the two linear superpositions of the Te/Se p_z orbitals in one unit cell, denoted as $\psi_1 = p_z^A + p_z^B$ and $\psi_2 = p_z^A - p_z^B$. The odd-parity $|\Gamma_6^-, \pm\frac{1}{2}\rangle$ band includes a significant contribution from the ψ_1 state, while the ψ_2 state mainly contributes to the even-parity bands 3 eV below the Fermi level. Now let's consider the contribution from the $\mathcal{H}_P$ Hamiltonian, which breaks inversion symmetry and thus can mix bands with different parities. From the second order perturbation, one can see that the asymmetric potential induced mixing can lead to a correction to the energy of the $|\Gamma_6^-, \pm\frac{1}{2}\rangle$ bands, given by

$$\Delta E_{\Gamma_6^-,\pm\frac{1}{2}} \propto \frac{|\langle\psi_1|\mathcal{H}_P|\psi_2\rangle|^2}{E_{\psi_1} - E_{\psi_2}} = \frac{|\langle p_z^A|\mathcal{H}_P|p_z^A\rangle - \langle p_z^B|\mathcal{H}_P|p_z^B\rangle|^2}{E_{\psi_1} - E_{\psi_2}} = \frac{4|V_g|^2}{E_{\psi_1} - E_{\psi_2}} > 0. \quad (39)$$

Here we use E_{ψ_1} and E_{ψ_2} to label the eigen-energy of the $|\Gamma_6^-, \pm\frac{1}{2}\rangle$ bands near the Fermi energy and the even-parity bands 3 eV below the Fermi level, respectively, and thus $E_{\psi_1} > E_{\psi_2}$. This perturbation calculation shows how the asymmetric potential can

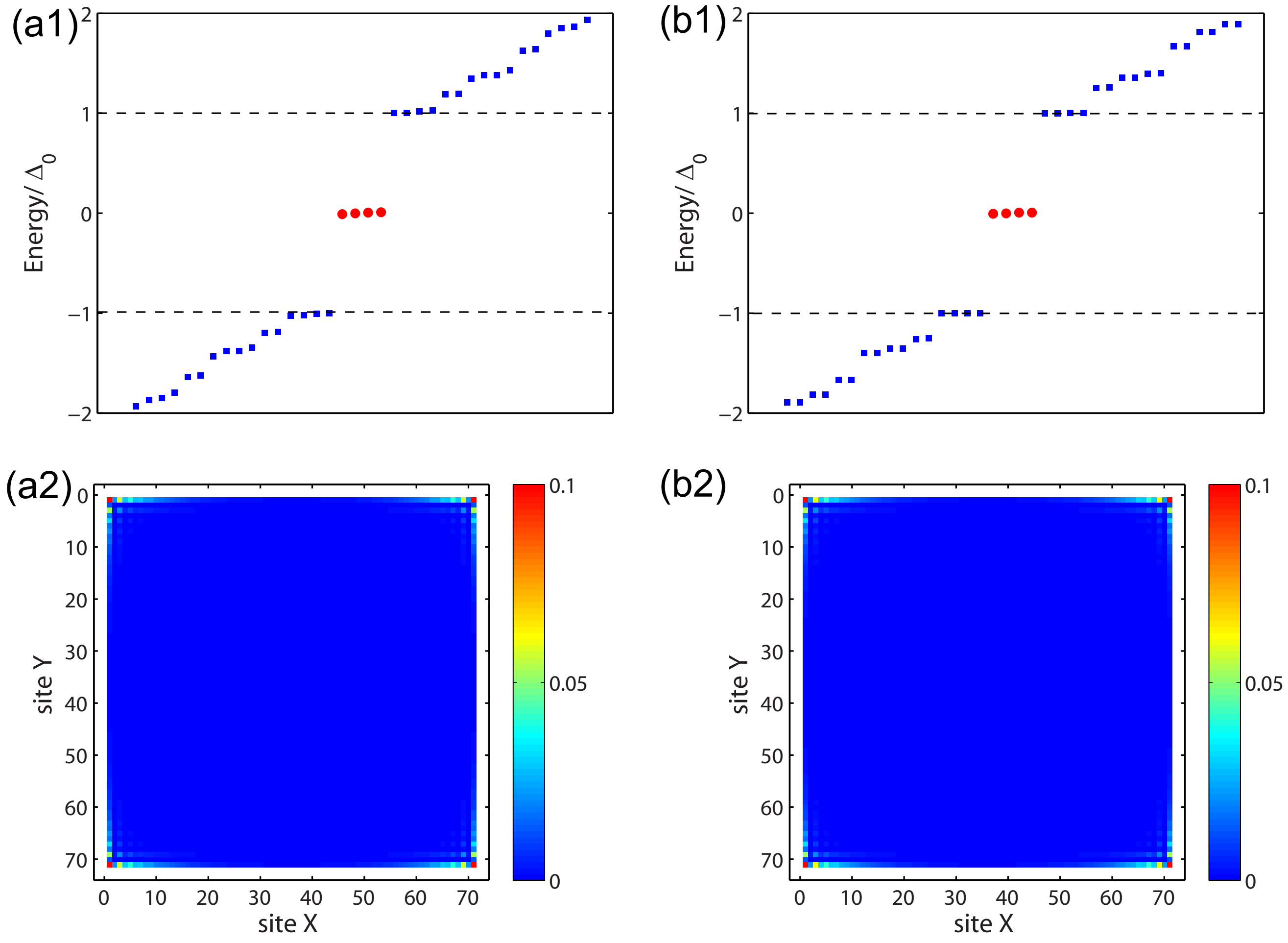

FIG. 5: (color online) Energy spectra and wavefunctions for MZM with potential disorder (a) and magnetic disorder (b). The adopted potential and magnetic disorder are uniform-distributed in [-W,W]. The corresponding strengths are $W = 0.1\Delta_0$ for potential disorder and $W = 0.1 g\mu_B|B| = 1.82$ meV for magnetic potential, respectively.

shift the eigen-energy of $|\Gamma_6^-, \pm\frac{1}{2}\rangle$ bands and lead a topological phase transition to drive the system into a topologically trivial phase, which discussed in Fig. 4 of the main text.

* Electronic address: cxl56@psu.edu

1 X. Wu, S. Qin, Y. Liang, H. Fan, and J. Hu, Phys. Rev. B **93**, 115129 (2016).
2 X. Wu, Y. Liang, H. Fan, and J. Hu, arXiv: 1603.02055 (2016).
3 J. Alicea, Rep.Prog. Phys. **75**, 076501 (2012).
4 Li, F. *et al*, Phys. Rev. B **91**, 220503 (2015).

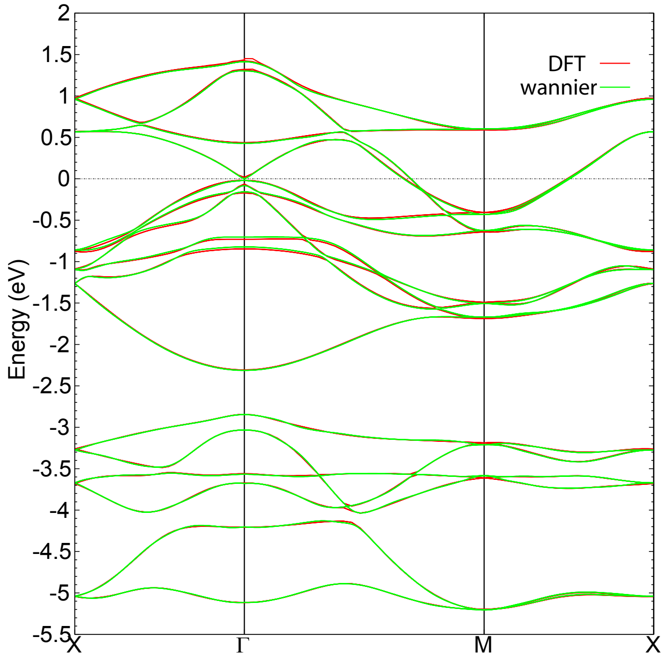

FIG. 6: (color online) Band structures for monolayer FeSe from DFT and maximum localized Wannier function method. The chemical potential is shifted up by 0.235 eV to model the electron doping from the substrate in experiments.
